# Relation of childhood diarrheal morbidity with the type of tube well used and associated factors of *Shigella sonnei* diarrhea in rural Bangladesh site of the Global Enteric Multicenter Study

**DOI:** 10.1186/s41182-019-0158-5

**Published:** 2019-05-02

**Authors:** Yasmin Jahan, Michiko Moriyama, Soroar Hossain, Md. Moshiur Rahman, Farzana Ferdous, Shahnawaz Ahmed, Sumon Kumar Das, Md. Iqbal Hossain, Abu Syed Golam Faruque, Tahmeed Ahmed, Mohammod Jobayer Chisti

**Affiliations:** 1 Graduate School of Biomedical & Health Sciences, Hiroshima University, Hiroshima-shi, Japan; 2 Graduate School of Comprehensive Human Sciences, University of Tsukuba, Tsukuba-shi, Japan; 3 Child Malnutrition Unit, Nutrition and Clinical Services Division, International Centre for Diarrhoeal Disease Research, Bangladesh (icddr,b), 68 Shaheed Tajuddin Ahmed Sarani, Mohakhali, Dhaka, 1212 Bangladesh; 4 Child Health Division, Menzies school of health Research, Northern Teritorry, Australia

**Keywords:** Bangladesh, *Shigella*, Shigellosis, Tube well water, Under-five children

## Abstract

**Background:**

Diarrheal disease still remains a major public health threat and is often associated with fatal outcome especially in children with shigellosis mostly in developing countries. This study aimed to determine the presence of any associations between drinking shallow tube well (STW) water and childhood shigellosis. A total of 1394 children aged 0–59 months who presented with moderate-to-severe diarrhea (MSD) in Kumudini Women’s Medical College and Hospital, Bangladesh, from December 2007 to March 2011 were enrolled into the study.

**Results:**

Among the study children, STW users often represented poor families (44% vs. 37%, *p* = 0.010); less often had household electricity (60% vs. 68%, *p* = 0.001) and cemented floor material (12% vs. 21%, *p* < 0.001); washed hand before eating (79% vs. 84%, *p* = 0.020); and had *Shigella sonnei* infections (7.8% vs. 13.1, *p* = 0.002) compared to deep tube well (DTW) water families (in bivariate analysis). After adjusting for covariates, a significant negative association was observed between childhood MSD episodes due to *Shigella sonnei* infections and the use of STW water (aOR 0.53, 95% CI 0.36, 0.79).

**Conclusions:**

An emergence of less severe *Shigella sonnei* has replaced relatively more severe *Shigella flexneri* among the MSD children from DTW-user families. However, more monitoring in terms of disease surveillance for changes in the distribution of *Shigella* serogroups and serotypes and its upsurges and antimicrobial susceptibility is essential.

## Background

Recently, global estimates attribute that in 2016, diarrhea accounted for 9% of 5.6 million deaths in children less than 5 years of age (U5s) worldwide making it the second leading cause of child mortality [1]. The highest rates of child mortality are in sub-Saharan Africa and Southeast Asia [1]. In 2013, shigellosis was responsible for 28,000–48,000 deaths annually among those under 5 years [2, 3].

*Shigella* transmission occurs via the fecal-oral route, person-to-person contact, household flies, infected water, and inanimate objects [4]. The minimal infectious dose can be transmitted directly from contaminated fingers since intermediate bacterial replication is not required to achieve the low infectious dose [2]. Shigellosis occurs predominantly in developing countries due to overcrowding and poor sanitation [4]. According to the Bangladesh Demographic and Health Survey (BDHS) report (2014), rural Bangladesh enjoys universal access to an improved source of drinking water (97%). A tube well is virtually the only source of drinking water (94%). Other infrequent sources of drinking water in rural Bangladesh are protected well (< 1%), rain water (< 1%), bottled water (< 1%), water piped into dwelling (< 1%), water piped to yard/plot (< 1%), and public tap/standpipe (< 1%) [5]. In the case of 74% of households, the drinking water source is located within the household premise, costs are affordable, and typically less than 45 m deep, it is known to be a shallow tube well (STW). In rural areas, because of distance and other contextual factors, more time is needed in collecting water for drinking purposes than urban Bangladesh. According to the BDHS (2014) report, one in five households spends less than 30 min on foot in two-way trips in collecting water for drinking purposes [5]. Other tube wells are found in agriculture fields, and available water is used for irrigation and household consumption by family members and called deep tube wells (DTW; ≥ 45 m deep). Studies have indicated that about 50% of the water samples collected from STW in Bangladesh was contaminated with human fecal organisms [6–8]. Due to their shallowness, STW water is prone to have contamination from leakage of neighborhood polluted water [6]. More generally, fecal contamination of shallow groundwater could possibly be one of the important reasons for the prevalence of diarrheal disease in Bangladesh [7, 8]. In many cases, immediate environmental conditions are unfavorable, e.g., the distance of tube wells from latrines or sewage-contaminated ponds or tanks may be insufficient to avoid the contamination of the well water with human-pathogenic bacteria. Tube wells have failed to protect against gastrointestinal diseases in Bangladesh, despite regular use of tube well water for drinking [9]. Recent studies in Bangladesh have demonstrated that up to 65% of tube wells can contain indicators of fecal contamination such as fecal/thermotolerant coliforms and *Escherichia coli* (*E. coli*); the level of contamination, however, is typically low [6–8, 10–14]. Fecal pathogens including rotavirus, adenovirus, *Shigella*, *Vibrio cholerae*, and *enterotoxigenic E. coli* have also been detected in tube well water [10–13]. Among the microbiological contamination, Bangladesh has the highest rates of shigellosis reported by two recent multi-country studies [15].

The species distribution of *Shigella* [*Shigella flexneri* (*S. flexneri*), *Shigella boydii* (*S. boydii*), *Shigella dysenteriae* (*S. dysenteriae*), and *Shigella sonnei* (*S. sonnei*)] varies globally. The first three are often prevalent in developing countries while *S. sonnei* (least virulent *Shigella* bacterium) is common in developed countries and usually causes a self-limiting febrile watery diarrhea [8, 16–18]. *Shigella boydii* causes disease of intermediate severity and is least common of the four. *S. flexneri* is less virulent than *S. dysenteriae* type 1 (the epidemic strain that causes severe life-threatening disease) but can also cause bloody diarrhea and abdominal cramps [17–19], whereas *S. flexneri* cases have been observed to report to the facility with increased number of days of the illness at home, higher numbers of episodes of diarrhea, and longer duration of mucoid/bloody diarrhea as well as hospitalization than *S. sonnei* [20].

Changing patterns in the distribution of *Shigella* serogroups and serotypes have been reported in Bangladesh [21]. In recent years, changes in *Shigella* serogroups, their geographical diversity, and emergence of *S. sonnei* have been reported in Bangladesh [21]. These changes have been indicated by researchers to be due to improved sanitary and hygienic practices, better living environment, improved nutritional status of children, and better access to safe drinking water. As shigellosis is a leading spectrum of diarrhea among the children presenting to the study sentinel health facility (SHC) in the present rural study community, this study aimed to determine any association between drinking STW water and childhood shigellosis [22] after adjusting for potential confounding impact of improved sanitary and hygienic practices, better living environment, improved nutritional status of children, and better access to safe drinking water. We hypothesized that because of the high proportion of households using STW water, there is an association between prevalence of childhood shigellosis particularly due to *S. sonnei* serogroup and STW water use in the Mirzapur community of rural Bangladesh.

## Methods

### Study site

For this secondary data analysis, relevant data were extracted from the database of the Global Enteric Multicenter Study (GEMS), Bangladesh site [22]. For this comparative study, all under-5 children from the STW water-using families comprised the study group and children from DTW-user households represented the comparison group [23]. The GEMS Bangladesh site was in a rural community of Bangladesh, located in Mirzapur sub-district of Tangail district, 60 km north of Dhaka, the capital city. The study had a backup of an ongoing demographic surveillance system (DSS). The sentinel health facility of the study was known as Kumudini Women’s Medical College and Hospital (KWMCH) (750 beds) which was situated in the middle of the study DSS area. All study participants (under-5 children with moderate-to-severe diarrhea (MSD) from the DSS area) were enrolled in the study in a sentinel health facility [22].

### Study design and enrollment of study participants

The GEMS study during December 2007 to February 2011 followed a case-control cohort design. Under-5 children, residents of the DSS catchment area, presenting with MSD (fulfilling at least one of the following criteria: sunken eyes, loss of skin turgor, intravenous rehydration administered or prescribed, dysentery—visible blood in loose stools, or admission to hospital with diarrhea or dysentery) within 7 days of onset of an acute illness (onset after ≥ 7 diarrhea-free days) constituted as GEMS cases. In this study, parents or primary caretakers of patients only with MSD underwent standardized interviews to solicit demographic, epidemiological, and clinical information at enrollment. Nutritional assessments (weight, length/height, and midupper arm circumference (MUAC)) were performed at the time of enrollment (before rehydration) and after rehydration, and *z*-scores were calculated and categorized as underweight (weight-for-age *z*-score < − 2), stunting (height-for-age *z*-score < − 2), and wasting (weight-for-height *z*-score < − 2) following the WHO guideline [2].

### Specimen collection and laboratory procedure

A single fresh stool (minimum of 3 g) was collected from each enrolled case child in the facility which within 1 h of passage was placed in a cold storage until delivery to the laboratory. Additionally, two rectal swabs for bacterial culture pending passage of the whole stool were obtained only when antibiotics were given to patients before stool was produced. All stool samples were shifted to the Clinical Microbiology Laboratory of International Centre for Diarrhoeal Disease Research, Bangladesh (icddr,b), Dhaka, as per standard guidelines. Bacterial pathogens [*Salmonella*, *Shigella*, *Campylobacter*, *Aeromonas* spp., *Vibrio cholerae*, and *Escherichia coli* (enterotoxigenic, enteropathogenic, and enteroaggregative)], viruses (rotavirus, norovirus, sapovirus, astrovirus, and adenovirus), and protozoa (*Entamoeba histolytica*, *Giardia intestinalis*, and *Cryptosporidium* spp.) were detected following standard laboratory methods [16].

### Data analysis

Statistical Package for Social Sciences (SPSS) Windows (Version 20, Chicago, IL) was used for data analysis, and Epi Info (Version 7.0) was used to calculate unadjusted odds ratios. Statistical analyses included descriptive as well as analytic methods. For the categorical variable of interest, the significance of differences was evaluated by chi-square (*χ*^2^) test. Odds ratios (ORs) were calculated to assess the association between STW water use and the independent variables of interest. ORs also indicated the strength of association; in addition to ORs, their 95% confidence intervals (CIs) were also estimated. Principal component was categorized and performed to determine wealth quintiles (by using household assets), assuming that factor loadings for certain household assets may vary through the years. Variables that were studied are construction material of the wall, roof, and floor of the house and household assets like radio, television, cell phone, and table. The wealth index was used as a measure of socio-economic status (SES) using information on household possessions. A weight was attached to each item from the first principal component. The households were classified into SES quintiles based on the wealth index: quintile (poor, lower middle, middle, upper middle, and rich).

Before performing logistic regression model, we also checked multicollinearity between independent variables using variance inflation factor (VIF). In the final model, the VIF values of all independent variables were less than 2 and the mean VIF was 1.17. To estimate ORs of several variables (selected on the basis of either statistical significance or biological importance), we used a multiple logistic regression model using forward elimination and taking tube well water use status as a dependent variable (coded as 1 = shallow tube well water use and 0 = deep tube well water use) and primary caretaker education (illiterate, i.e., no formal schooling = 1, literate = 0); number of living room in the household (1–4 = 1, > 4 = 0); floor material (earth/soil/non-cemented = 1, cemented = 0); household electricity (no = 1, yes = 0); treatment of drinking water (no = 1, yes = 0); treatment method (1 = filter through cloth, 0 = use of water filter); container observed to be covered (no = 1, yes = 0); hand wash use material (water only = 1, water and soap = 0); hand washing before eating (no = 1, yes = 0); cooking (no = 1, yes = 0); nursing (no = 1, yes = 0); after defecation (no = 1, yes = 0); place of feces disposal (traditional pit toilet = 1, pour flush = 0); nutritional status (stunting = < − 2.00 = 1, no stunting = 0; underweight = < − 2.00 = 1, no underweight = 0; wasting < − 2.00 = 1, no wasting = 0); wealth quintile (poor = 1, rich = 0); age (12–59 months = 1, 0–11 months = 0); and sex (girl = 1, boy = 0) as independent variables. The *P* value cutoff of 0.1 (the significance level of variables for inclusion) was considered adequate to prevent residual confounding in the forward step-wise logistic regression model [23–25]. Statistical significance to remain in the final multivariable model was set at < 0.05.

## Results

**Fig. 1:**
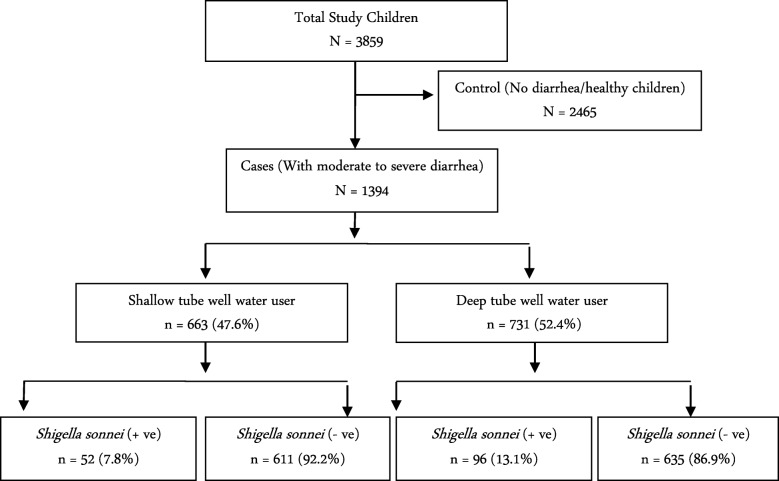
Study profile of enrolled children (cases with moderate-to-severe diarrhea)

Of the overall 3859 children enrolled during the study period, 1394 had MSD (and their data were analyzed in this study) and 2465 had no diarrhea. Among the MSD children, 47.6% families (*n* = 663) were a user of STW, while 52.4% (*n* = 731) represented DTW water-user families (Fig. 1).

Severity of diarrheal illness and dehydration status were found similar in STW- and DTW-user families (Table 1). The study classified all the children into three age groups: 550 children aged 0–11 months, 476 children aged 12–23 months, and 368 children aged 24–59 months. Age and nutritional status of study children were identical in two groups. Among the MSD children, 814 were boys and 580 were girls. We did not find any association between gender and tube well water use. STW users often represented poor families than DTW users. The number of sleeping rooms in the house, presence of household electricity, cemented floor material, and practice of washing hands before eating and nursing were observed significantly less often among the STW-using household members than their counterparts from DTW-user families (Table 2). *Shigella sonnei* infection was detected less frequently in stool specimens of children from STW-user families than DTW-user family (7.8% vs. 13.1%; *p* = 0.002) (Table 3).

**Table 1:** Enrollment features of study children

Variables	Shallow tube well water user (*N* = 663), *n* (%)	Deep tube well water user (*N* = 731), *n* (%)	*p* value*
Sunken eye	108 (16.3)	112 (15.3)	0.673
Loss of skin turgor	35 (5.3)	26 (3.6)	0.150
Intravenous rehydration	77 (11.6)	88 (12.0)	0.871
Dysentery	478 (72.1)	551 (75.4)	0.183
Hospitalized	145 (21.9)	138 (18.9)	0.186

*p value stands for chi-square tests which were performed to examine the presence of significant association

**Table 2:** Characteristics of under-5 moderate-to-severe diarrheal children from the two types of tube well water-user families

Variables	Shallow tube well (*N* = 663), *n* (%)	Deep tube well (*N* = 731), *n* (%)	Unadjusted OR (95% CI)	*p* value
Age (month)				
0–11	264 (39.8)	286 (39.1)	1.03 [0.83, 1.27]	0.957
12–23	226 (34.1)	250 (34.2)	0.99 [0.79, 1.24]	
24–59	173 (26.1)	195 (26.7)	0.97 [0.76, 1.23]	
Gender				
Girl	277 (41.8)	303 (41.5)	1.01 [0.81, 1.25]	0.944
Boy	386 (58.2)	428 (58.5)		
Maternal education				
Illiterate	79 (11.9)	78 (10.7)	1.13 [0.81, 1.57]	0.516
Literate	584 (88.1)	653 (89.3)		
Sleeping room				
1–4	630 (95.0)	666 (91.1)	1.86 [1.21, 2.87]	0.005*
5–10	33 (5.0)	65 (8.9)		
Floor				
Earth	582 (87.8)	578 (79.1)	1.90 [1.41, 2.54]	< 0.001*
Cemented	81 (12.2)	153 (20.9)		
Electricity				
No	268 (40.4)	233 (31.9)	1.45 [1.16, 1.80]	0.001*
Yes	395 (59.6)	498 (68.1)		
Water trips daily				
1–4	348 (53.0)	386 (52.8)	0.99 [0.80, 1.22]	0.994
5–26	309 (47.0)	345 (47.2)		
Treating method				
Through cloth	628 (94.7)	721 (98.6)	0.24 [0.12, 0.50]	< 0.001*
Filter	35 (5.3)	10 (1.4)		
Treat drinking water				
No	615 (92.8)	718 (98.2)	0.23 [0.12, 0.43]	< 0.001*
Yes	48 (7.2)	13 (1.8)		
Type of container				
Wide mouth container	633 (99.5)	700 (99.4)	1.20 [0.26, 5.40]	1.000
Narrow mouth container	3 (0.5)	4 (0.6)		
Container covered				
No	363 (57.1)	384 (54.5)	1.10 [0.89, 1.37]	0.381
Yes	273 (42.9)	320 (45.5)		
Hand wash use				
Water with ash and mud	88 (13.3)	84 (11.5)	1.17 [0.86, 1.62]	0.353
Water and soap only	575 (86.7)	647 (88.5)		
Hand washing practice				
Before eating				
No	139 (21.0)	117 (16.0)	1.39 [1.06, 1.82]	0.020*
Yes	524 (79.0)	614 (84.0)		
Before cooking				
No	292 (44.0)	328 (44.9)	0.96 [0.78, 1.19]	0.797
Yes	371 (56.0)	403 (55.1)		
Before nursing				
No	506 (76.3)	521 (71.3)	1.29 [1.02, 1.65]	0.038*
Yes	157 (23.7)	210 (28.7)		
After defecation				
No	108 (16.3)	122 (16.7)	0.97 [0.73, 1.29]	0.897
Yes	555 (83.7)	609 (83.3)		
Fecal dispose				
Traditional pit toilet	368 (55.5)	438 (59.9)	0.83 [0.67, 1.03]	0.107
Pour flush toilet	295 (44.5)	293 (40.1)		
Nutritional status				
% stunting	156 (23.5)	179 (24.5)	0.95 [0.74, 1.21]	0.722
% underweight	221 (33.3)	232 (31.7)	1.07 [0.86, 1.35]	0.563
% wasting	141 (21.3)	154 (21.1)	1.01 [0.78, 1.31]	0.980
Wealth quintile				
Poor	291 (43.9)	270 (36.9)	1.33 [1.08, 1.66]	0.010*
Rich	372 (56.1)	461 (63.1)		

*OR* odds ratio, *CI* confidence interval, *p value statistically significant at < 0.05

**Table 3:** Pathogen distribution among under-5 moderate-to-severe diarrheal children from the two types of tube well water user families

Pathogen variables	Shallow tube well water user (*N* = 663), *n* (%)	Deep tube well water user (*N* = 731), *n* (%)	Unadjusted ORs (95% CI)	*p* value
*Shigella* spp.				
Yes	266 (40.1)	325 (44.5)	0.84 [0.68, 1.04]	0.113
No	397 (59.9)	406 (55.5)		
*S. flexneri*				
Yes	195 (29.3)	206 (27.8)	1.06 [0.84, 1.34]	0.654
No	468 (70.7)	525 (72.2)		
*S. sonnei*				
Yes	52 (7.8)	96 (13.1)	0.56 [0.39, 0.80]	0.002*
No	611 (92.2)	635 (86.9)		
*S. boydii*				
Yes	7 (1.1)	15 (2.1)	0.43 [0.17, 1.05]	0.364
No	656 (98.9)	716 (97.9)		
*S. dysenteriae*				
Yes	4 (0.6)	10 (1.4)	0.44 [0.14, 1.46]	0.246
No	659 (99.4)	721 (98.6)		
*V. cholerae*				
Yes	10	9	0.99 [0.40, 2.46]	1.000
No	721	654		
*Salmonella*				
Yes	17	25	1.65 [0.88, 3.07]	0.120
No	714	638		
ETEC				
Yes	27 (4.1)	38 (5.2)	0.77 [0.47, 1.28]	0.385
No	693 (94.8)	636 (95.9)		
EPEC				
Yes	69	65	1.19 [0.83, 1.69]	0.385
No	594	666		
EAEC				
Yes	155 (23.4)	178 (24.4)	0.95 [0.74, 1.21]	0.717
No	508 (76.6)	553 (75.6)		
*Aeromonas*				
Yes	170 (25.6)	186 (25.4)	1.01 [0.79, 1.28]	0.982
No	493 (74.4)	545 (74.6)		
*Campylobacter*				
Yes	115 (17.3)	131 (17.9)	0.96 [0.73, 1.26]	0.833
No	548 (82.7)	600 (82.1)		
Rotavirus				
Yes	113 (17.0)	106 (14.5)	1.21 [0.91, 1.62]	0.219
No	550 (83.0)	625 (85.5)		
Norovirus				
Yes	44 (6.6)	59 (8.1)	0.81 [0.540, 1.21]	0.358
No	619 (93.4)	672 (91.9)		
Adenovirus				
Yes	20 (3.0)	30 (4.0)	0.73 [0.41, 1.29]	0.344
No	643 (97.0)	701 (96.0)		
Astrovirus				
Yes	8 (1.2)	9 (1.2)	0.98 [0.37, 2.55]	1.000
No	655 (98.8)	722 (98.8)		
Sapovirus				
Yes	10 (1.5)	9 (1.2)	1.22 [0.49, 3.04]	0.830
No	653 (98.5)	722 (98.8)		
*Cryptosporidium*				
Yes	49 (7.4)	49 (6.7)	1.11 [0.74, 1.67]	0.692
No	614 (92.6)	682 (93.3)		
*Giardia*				
Yes	51 (7.7)	55 (7.7)	1.02 [0.69, 1.52]	0.986
No	612 (92.3)	676 (92.5)		
*E. histolytica*				
Yes	51 (7.7)	42 (5.7)	1.37 [0.89, 2.08]	0.178
No	612 (92.3)	689 (94.3)		

*OR* odds ratio, *CI* confidence interval, *p value statistically significant at < 0.05

The association of *S. sonnei* infection in STW water user group was further ascertained by regression analysis controlling for other variables. And we observed that children with *S. sonnei* infection were negatively associated with STW water use (aOR 0.55, 95% CI 0.37, 0.80) (Table 4). *S. sonnei* infection was also found positively associated with older children (age 12–59 months) (aOR 2.34, 95% CI 1.84, 2.97), not covering drinking water container (aOR 1.45, 95% CI 1.00, 2.10), and non-use of soap during hand washing (aOR 2.17, 95% CI 1.34, 3.49) (Table 4).

**Table 4:** Association between *Shigella sonnei* infection, tube well use, and other factors in rural Mirzapur (number of subjects: *S. sonnei* + ve, *n* = 148; *S. sonnei* − ve, *n* = 1246, total, *N* = 1394)

Variables	Unadjusted ORs	95% CI	Adjusted ORs	95% CI	*p* value*
Shallow tube well	0.56	0.39–0.80	0.55	0.37–0.80	0.002*
Age (12–59 months)	5.04	3.07–8.27	2.34	1.84–2.97	< 0.001*
Gender (girl)	0.93	0.65–1.32	0.89	0.62–1.28	0.553
Wealth index (poor)	0.57	0.39–0.83	0.80	0.47–1.36	0.419
No maternal education	0.42	0.20–0.87	0.51	0.24–1.16	0.093
Household sleeping room (< 4)	0.58	0.33–1.02	0.75	0.40–1.40	0.370
Household floor (earth/mud)	0.73	0.48–1.12	0.94	0.58–1.53	0.819
No household electricity	0.56	0.38–0.82	0.70	0.41–1.18	0.188
No treatment of drinking water	0.77	0.36–1.66	0.67	0.30–1.52	0.344
Did not cover drinking water container	1.28	0.90–1.82	1.45	1.00–2.10	0.048*
Did not use soap during hand washing	1.78	1.14–2.79	2.17	1.34–3.49	0.001*
Did not wash hand before eating	0.89	0.56–1.40	1.04	0.64–1.68	0.852
Did not wash hand before cooking	0.94	0.67–1.33	0.90	0.62–1.29	0.569
Did not wash hand before nursing	0.92	0.63–1.35	0.88	0.58–1.33	0.551
Did not wash hand after defecation	0.67	0.40–1.12	0.58	0.34–1.01	0.057
Child feces disposal (pour flush toilet)	1.26	0.88–1.79	1.06	0.71–1.58	0.751
Weight for height *z*-score (wasting)	1.07	0.71–1.62	1.02	0.57–1.74	0.995
Height for age *z*-score (stunted)	0.85	0.56–1.29	0.89	0.53–1.47	0.654
Weight for age *z*-score (underweight)	0.89	0.62–1.29	0.78	0.44–1.38	0.406

*OR* odds ratio, *CI* confidence interval

*p values are of adjusted ORs and statistically significant at < 0.05

## Discussion

We hypothesized that because of higher proportion households using STW water, there would be an association between greater prevalence of childhood shigellosis particularly due to the *S. sonnei* serogroup among STW water-user families in the Mirzapur community of rural Bangladesh. However, our results refuted the hypothesis. We know that shigellosis is typically associated with poverty, poor hygiene, and crowded living conditions in developing countries, as well as an important contributor to childhood malnutrition. Shigellosis can occur even after ingestion of low inoculums (10–100 organisms) [26]. Usually, it is transmitted by fecal-oral route due to contamination of the hands, food, and water with infected feces. Incidence is higher in children 1–5 years of age, presumably as good personal hygiene is much more difficult to achieve in young children particularly who have not yet acquired specific immunity [27]. In our study, we found poor hygiene practices especially covering of water container, use of hand washing substances, and children from poor families are highly vulnerable to childhood shigellosis. Alternatively, we did not find any significant association between *S. flexneri*, *S. boydii*, and *S. dysenteriae* infections and STW water use in children presenting with MSD episodes to the study sentinel health center. Moreover, we also did not find any noteworthy relationship between non-shigella species (*Salmonella*, *Campylobacter*, *Aeromonas spp*., *V. cholerae*, *E.coli*, rotavirus, astrovirus, adenovirus, norovirus, sapovirus, *Giardia lamblia*, *Cryptosporidium*, and *E. histolytica*) and STW water. Surprisingly, we observed a protective relationship between *S. sonnei* infections and STW water use for the first time in Bangladesh. Additionally, tube well water use impacted positively by demonstrating an emergence of less severe *Shigella* strain known to be as *S. sonnei* replacing a more severe *Shigella* strain called as *S. flexneri* among the MSD children from DTW-using families*.* Bangladesh has made adequate progress over recent years, and despite poverty, the economy is growing by about 6% each year. Such gross economic improvement has demonstrated concurrent positive behavioral changes like more access to safe drinking water.

*Shigella* spp. are dynamic and able to survive under diverse environmental conditions [28]. Shigellosis due to *S. sonnei* has been previously reported to occur less often among people living in developing countries. In this study, we revealed isolation of *S. sonnei* more commonly from MSD children reporting from DTW water-user families than STW water users. Infections caused by *S. sonnei* have become more common than those due to *S. flexneri* in a population that are reasonably well-off and living with improved water and sanitation practices. Such emergence of *S. sonnei* has also been reported even in Bangladesh in recent years [29, 30]. The relation between upsurge of *S. sonnei* infections among the MSD children and economic development may be explained by the less exposure of individuals from developing countries including Bangladesh to *Plesiomonas shigelloides* (*P. shigelloides*) in recent years [15].

*P. shigelloides* is a gram-negative bacterium often found in surface water that shares antigens with *S. sonnei*, and *P. shigelloides* has been a frequently isolated enteric pathogen from young diarrhea children aged less than 2 years old [31–33]. It is well recognized that exposure to *P. shigelloides*, through contaminated drinking water may immunize people to *S. sonnei* in the developing countries where people are often exposed to contaminated water drink [30]. With economic progress and accompanying water quality improvements (as a result, there is less exposure to *P. shigelloides*), susceptibility to *Shigella sonnei* in this population may have increased [28, 30]. The gross economic improvement in Bangladesh might have a positive impact with an increase in the use of DTW and bottled water, and the individuals in general have increasing access to improved piped water supply regularly in urban areas than in rural areas [21]. The changing trend as observed by the emergence of *Shigella sonnei* may be a reflection of this shifting phenomenon. However, such changes may require more monitoring in terms of disease surveillance for diversity in distribution of serogroups as well as serotypes, upsurges of cases of shigellosis, and antimicrobial susceptibility.

In this study, our strengths were the pre-set inclusion criteria for enrollment of children into the study which is undergoing fairly rapid economic development; identification of pathogens following standardized laboratory methods; and use of a large data set for this analysis. However, the study limitations were the lack of information like whether the areas of DTW were used to get often flooded, toilets of STW-using families were more hygienic, or garbage disposal areas were near the DTW than STW as well as water storing behavior of the family members of DTW users and so on. Moreover, our observations were based only on children with MSD and attending the KWMCH which might not represent the general population in a community including those with less severe disease and those who did not report to the sentinel health center.

## Conclusions

Our findings suggest that the emergence of less severe *Shigella sonnei* has replaced relatively more severe *Shigella flexneri* among the moderate-to-severe diarrhea children from DTW-user families. These observations signify the impact of gross economic improvement in Bangladesh and concurrent behavioral changes—more access to safe drinking water which impacted positively by demonstrating the emergence of *Shigella sonnei* replacing the *Shigella* strain known as *Shigella flexneri.* Therefore, further interventions are needed in the domestic domain to reduce any spread of shigellosis by water treatment and hygiene practices.

## Abbreviations

DSS: Demographic surveillance system
DTW: Deep tube well
GEMS: Global Enteric Multicenter Study
icddr,b: International Centre For Diarrhoeal Disease Research, Bangladesh
KWMCH: Kumudini Women’s Medical College and Hospital
MSD: Moderate-to-severe diarrhea
MUAC: Midupper arm circumference
SHC: Sentinel health facility
STW: Shallow tube well
WHO: World Health Organization
